# Immunization of N terminus of enterovirus 71 VP4 elicits cross-protective antibody responses

**DOI:** 10.1186/1471-2180-13-287

**Published:** 2013-12-10

**Authors:** Miao Zhao, Yu Bai, Wei Liu, Xiangqian Xiao, Yuming Huang, Shan Cen, Paul KS Chan, Xin Sun, Wang Sheng, Yi Zeng

**Affiliations:** 1 College of Life Science and Bioengineering, Beijing University of Technology, 100, Pingleyuan, Chaoyang District, Beijing 100124, PR China; 2 Research Center for Life Science, Beihua University, 3999, Binjiangdong Road, Jinlin, Jilin Province 132013, PR China; 3 Department of Neurology, Beijing Ditan Hospital, Capital Medical University, Beijing PR China; 4 Department of Virology, Institute of Medicinal Biotechnology, Chinese Academy of Medical Science, Beijing PR China; 5 Department of Microbiology, Chinese University of Hong Kong, Hong Kong PR China

**Keywords:** Enterovirus 71, Vaccine, VP4, Peptide, Chimeric virus-like particle

## Abstract

**Background:**

Enterovirus 71 (EV71) is major cause of hand, foot and mouth disease. Large epidemics of EV71 infection have been recently reported in the Asian-Pacific region. Currently, no vaccine is available to prevent EV71 infection.

**Results:**

The peptide (VP4N20) consisting of the first 20 amino acids at the N-terminal of VP4 of EV71 genotype C4 were fused to hepatitis B core (HBcAg) protein. Expression of fusion proteins in *E. coli* resulted in the formation of chimeric virus-like particles (VLPs). Mice immunized with the chimeric VLPs elicited anti-VP4N20 antibody response. *In vitro* microneutralization experiments showed that anti-chimeric VLPs sera were able to neutralize not only EV71 of genotype C4 but also EV71 of genotype A. Neonatal mice model confirmed the neutralizing ability of anti-chimeric VLPs sera. Eiptope mapping led to the identification of a “core sequence” responsible for antibody recognition within the peptide.

**Conclusions:**

Immunization of chimeric VLPs is able to elicit antibodies displaying a broad neutralizing activity against different genotypes of EV71 *in vitro*. The “core sequence” of EV71-VP4 is highly conserved across EV71 genotypes. The chimeric VLPs have a great potential to be a novel vaccine candidate with a broad cross-protection against different EV71 genotypes.

## Background

Human enterovirus 71 is a non-enveloped RNA virus of the *Picornaviridae* family. The virion is around 30 nm in diameter containing a single-stranded positive-sense RNA genome of approximately 7500 nucleotides [1-3]. The whole genome is translated into a single large polyprotein that can be subsequently processed by protease digestion to produce four capsid subunit proteins, VP1 to VP4 and other nonstructural proteins. The icosahedral capsid is composed of 60 sets structural proteins (VP1 to VP4). It has been shown that VP1-3 form a pseudo T = 3 icosahedral capsid that are located on the surface of viral capsid [4]. VP4 is located inside, which is approximately 70 amino acids in length and is myristoylated at the N terminus [5,6]. Crystallographic analysis showed that the mature EV71 virus is structurally similar to other enteroviruses [7].

EV71 and coxsackievirus A16 (CA16) have been identified as the two major etiological agents of hand, foot and mouth disease (HFMD) [8,9]. Large outbreaks of HFMD have recently been reported in the Asia-Pacific region, which is becoming a common acute viral disease in these areas and posing a serious health threat to children [10-13]. While HFMD is usually mild and self-limiting, it may lead to severe neurological complications and even death [14,15]. However, no effective vaccine is yet available to prevent EV71 infection.

The evidence that maternal mice vaccinated with the EV71 virus-like particles (VLPs) can confer protection to neonatal mice against lethal challenge reveals an essential role of neutralizing antibody in the protection against infection [3]. To determine the immunodominant epitopes of EV71 capsid protein, antisera generated from animals immunized with formalin-inactivated EV71 vaccine were screened against a set of overlapping synthetic peptides covering the entire sequences of VP1, VP2 and VP3 of EV71. Several linear immunodominant neutralization epitopes have been successfully identified in VP1 and VP2 proteins [16-20]. Numerous studies reported that synthetic peptides containing neutralizing epitope of VP1 elicited neutralizing antibody response and protected neonatal mice against lethal challenges [17-20]. Therefore, the epitope-based vaccine has a great potential to be a successful vaccine to prevent EV71 infection.

In the present study, the peptide consisting of N-terminal residues 1–20 of EV71 VP4 of genotype C4 was fused to hepatitis B core antigen (HBcAg) and expressed in *E. coli*. The resulting fusion proteins were able to spontaneously assemble into chimeric VLPs, which elicited virus-neutralizing antibody response. We further identified a highly conserved linear neutralizing epitope in the N-terminus of EV71 VP4 by epitope mapping experiments. Our results suggest that chimeric HBcAg particles carrying a neutralizing epitope of EV71 VP4 could be a promising vaccine candidate against EV71 infection.

## Results

### Generation of chimeric particles carrying the peptide VP4N20

**Figure 1:**
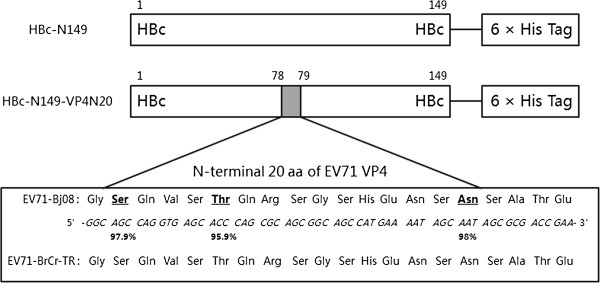
**Schematic presentation of the chimeric HBcAg protein construct.** The shaded box represents the N-terminal 20 a.a. of VP4 of Bj08 and BrCr-TR. Italics letters indicate nucleotide sequences, and the percentages indicate the degree of conservation among the 100 strains of EV71 from Asia.

The gene sequence and amino acid sequence of peptide VP4N20 as well as its insertion position in HBcAg are shown in Figure 1. The plasmid vector pET22b (+) (Novagen) encodes a six-histidine tag at the C-terminal region of recombinant proteins for convenient purification by affinity chromatography as well as expression analysis by Western-blot. A carboxyl-terminally truncated HBcAg protein (149 aa, HBc-N149) and a fusion protein (HBc-N149-VP4N20) were expressed in *E. coli*, respectively.

**Figure 2:**
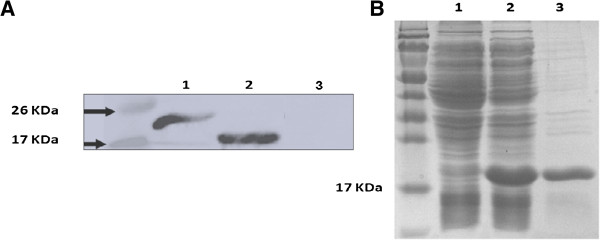
**Protein expression and purification.** The expression of HBc-N149 and HBc-N149-VP4N20 protein was detected by Western blot. **(A)** Lane 1: HBc-N149-VP4N20. Lane 2: HBc-N149. Lane 3: Negative control. The protein purification was visualized by SDS-PAGE. **(B)** Lane 1: Uninduced bacteria expressing HBc-N149-VP4N20; Lane 2: Induced bacteria expressing HBc-N149-VP4N20; Lane 3: Purified HBc-N149-VP4N20.

**Figure 3:**
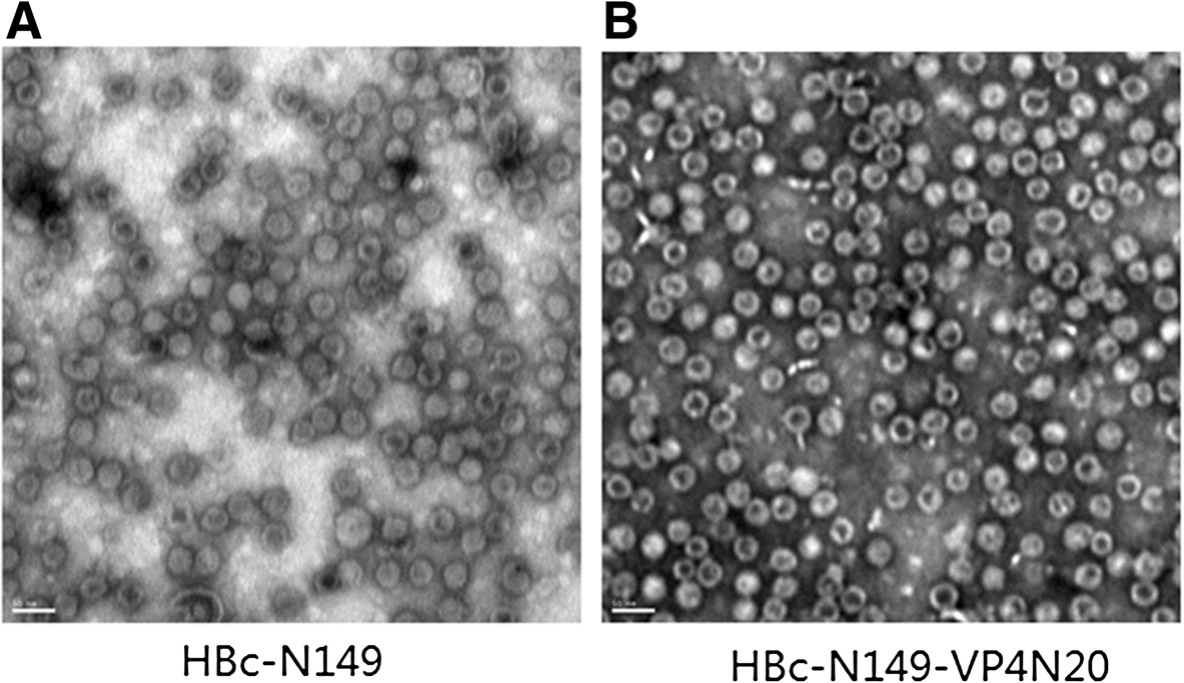
**Electron microphotographs of HBc**-**N149 and HBc**-**N149**-**VP4N20 particles. (A)** Particles assembled from HBc-N149. **(B)** Chimeric particles assembled from HBc-N149-VP4N20. Size bar: 50 nm.

The efficient expression of both proteins was demonstrated by Western-blot after IPTG induction (Figure 2A). They were further purified using Ni Sepharose column. The purity of proteins was evaluated by densitometric analysis after staining with Coomassie blue and the representative samples of expressed proteins were shown in Figure 2B. Since HBcAg protein can form particles both *in vivo* and *in vitro*, we then investigated whether the recombinant proteins can form particles. Electron microscopy analysis showed that both HBc-N149 and HBc-N149-VP4N20 proteins were able to efficiently form particles with the size around 25–30 nm (Figure 3). The results suggest that the chimeric proteins can self-assemble to form VLPs.

### Chimeric particle immunization elicited VP4N20 specific antibody responses in mice

**Figure 4:**
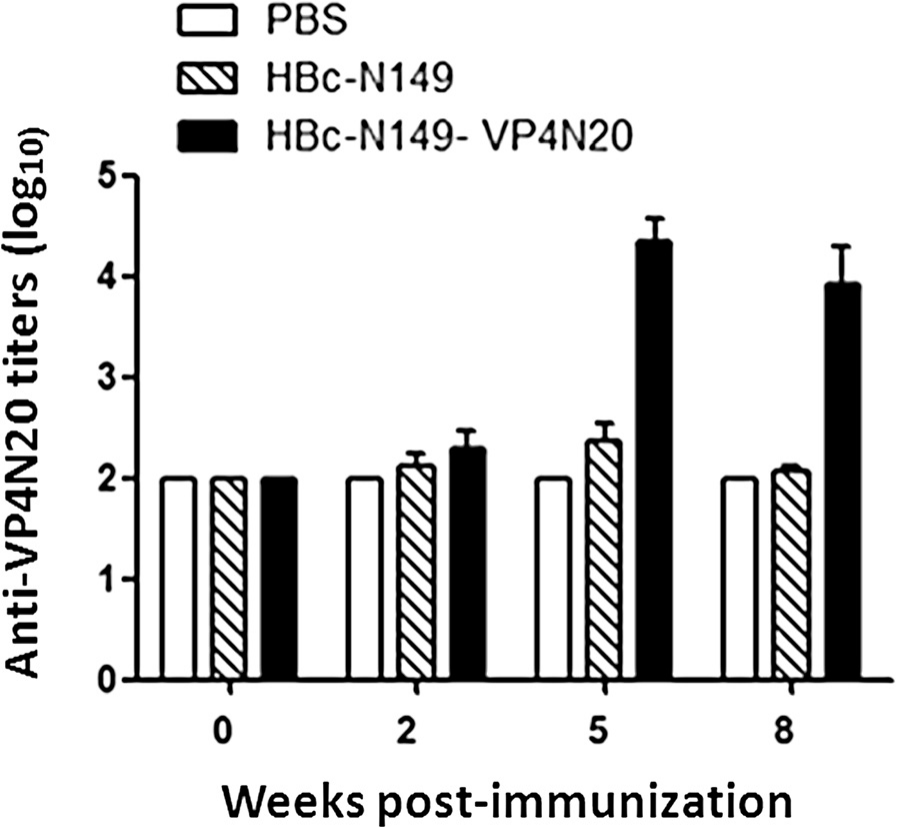
**Kinetics of antibody titer development in mice following immunization.** Mice were immunized twice with 100 μl preparation containing 5 μg of different proteins on week 0 and 3, respectively, and were bled before immunization and at week 2, 5, 8 weeks after immunization for serological tests. BSA-VP4N20 was used to coat EIA plates to detect VP4N20-specific antibodies. Each bar represents the mean reciprocal log10 endpoint titers and standard error.

To determine whether chimeric HBcAg particles were capable of eliciting anti-VP4N20 antibody, female BALB/c mice were immunized i.m. with either purified chimeric VLPs (HBc-N149-VP4N20) or HBcAg VLPs (HBc-N149) and received booster injections 3 weeks later. Mice immunized with PBS were used as negative controls. The immunized animals were bled at week 0, 2, 5, 8 for serological analysis. The results showed that anti-VP4N20 antibody became detectable in chimeric VLPs-immunized mice at 2 weeks after inoculation. The titers were enhanced by booster injection and reached a maximum at week 5. No anti-VP4N20 antibody response was detected in the HBcAg VLPs -immunized group and the PBS group. Our results indicated that chimeric particles were able to induce anti-VP4N20 immune responses (Figure 4).

### Chimeric VLPs were able to induce neutralizing antibodies against EV71

**Figure 5:**
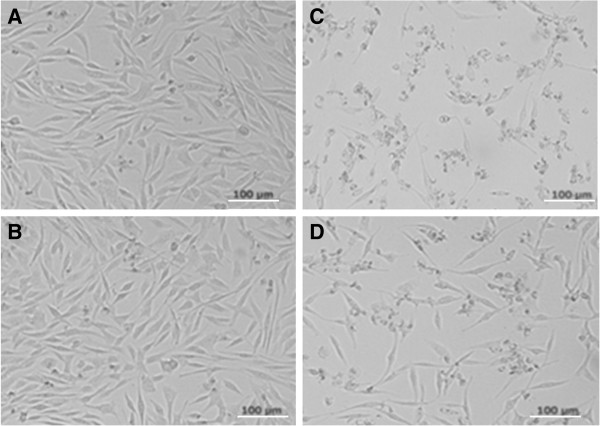
**Microneutralization assay results.** The virus/antiserum mixtures were added into RD cells and incubated at 37°C. After 7 days, the cells were observed to evaluate the appearance of cytopathic effects (CPEs). **(A)** Uninfected cells. **(B)** EV71-bound cells were treated with the anti-HBc-N149-VP4N20 sera. **(C)** EV71-infected cells. **(D)** EV71-bound cells were treated with the anti-HBc-N149 sera.

**Figure 6:**
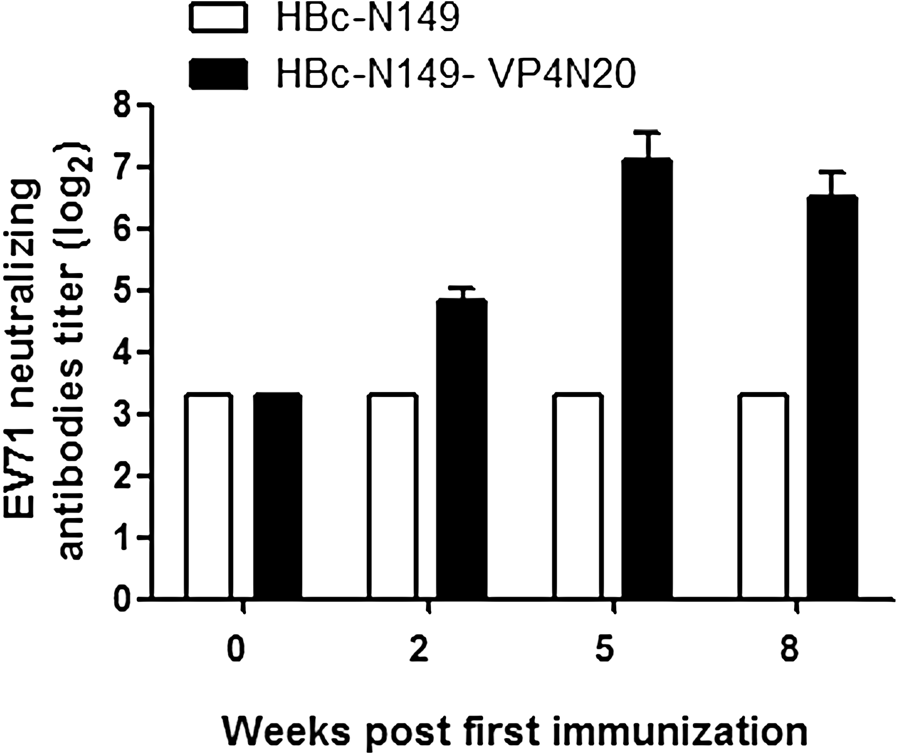
**Kinetics of neutralizing antibodies to EV71 following immunization.** Neutralizing antibodies in the sera of immunized mice to EV71 were measured by in vitro microneutralization assay. The neutralizing antibody titer was defined as the highest serum dilution that prevented the occurrence of cytopathic effects. Each bar represents the mean reciprocal log2 endpoint titers and standard error.

To evaluate whether the chimeric VLPs could induce neutralizing antibodies against EV71, sera from immunized mice were tested for the ability to neutralize live EV71 *in vitro*. EV71 (genotype C4) and a variant of the prototype strain of EV71, BrCr-TR (genotype A) were used for *in vitro* neutralization assay. As shown in Figure 5, the sera from the group immunized with chimeric VLPs were able to neutralize EV71 (Bj08 strain) and prevented RD cells from developing cytopathic effects. The highest neutralizing titre of around 1.36 × 10^2^ was obtained at week 5 post-immunization (Figure 6), which was consistent with the antibody profile as shown in Figure 4. However, anti-chimeric VLPs sera had a neutralizing activity against EV71 of type A (BrCr-TR) with a neutralization titre similar to that against Bj08 strain (data not shown). Amino acid sequence alignment show that the N-terminal sequence of the Bj08 VP4 is identical to that of BrCr-TR (Figure 1). Compared to chimeric particles, HBcAg particles failed to induce neutralizing antibody responses against EV71 (Bj08 strain) (Figure 5) as well as EV71 BrCr-TR strain (data not shown). Our results indicate that immunization of chimeric VLPs can elicit neutralizing antibody responses against EV71 and the sera exhibit a cross-neutralizing activity against EV71 strains belonging to different genotypes *in vitro*.

### Neonatal mice as a model to verify in vitro neutralizing ability of chimeric VLP-immunized sera

**Figure 7:**
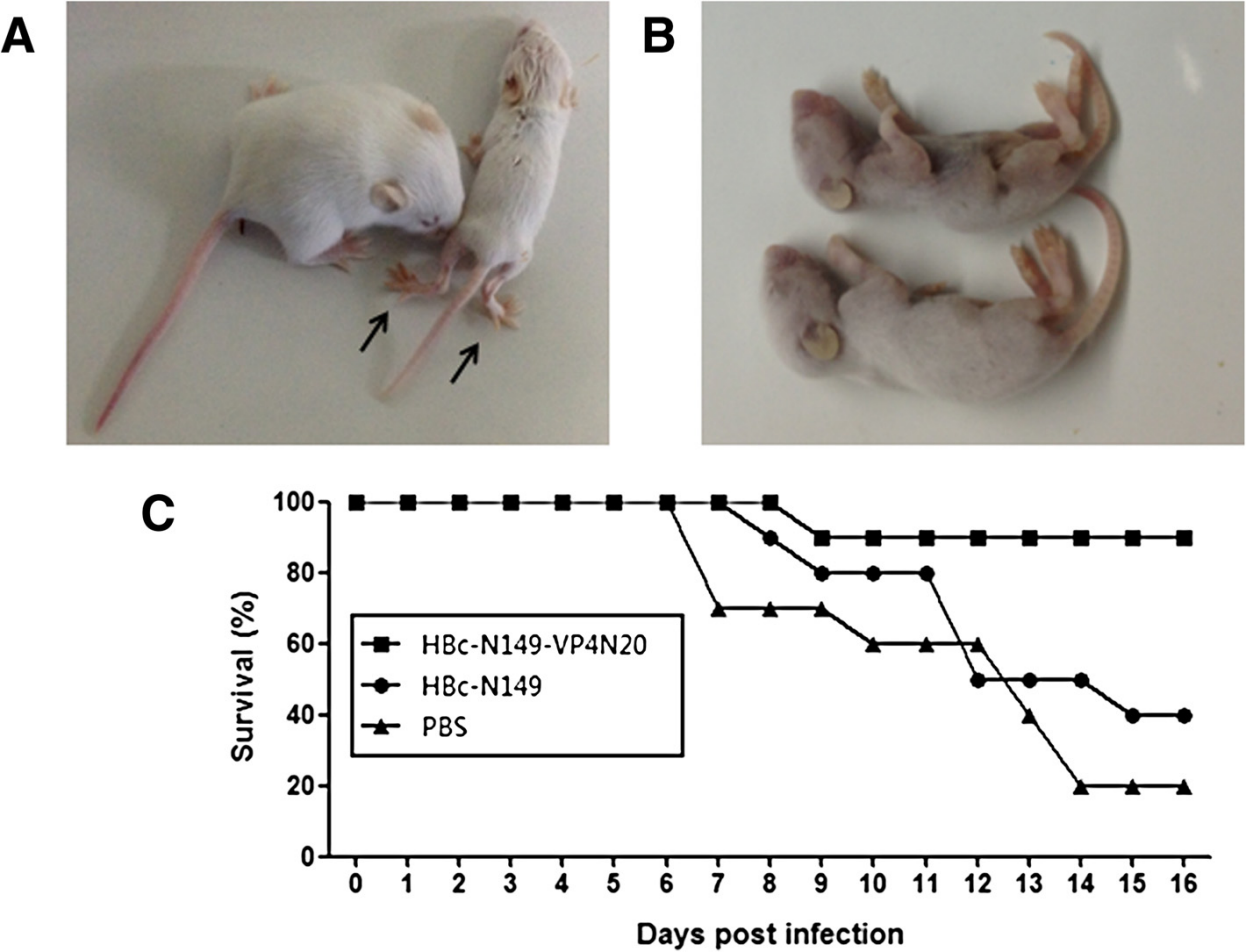
**Neonatal mice as a model to assess in-vitro neutralizing effects of anti-sera.** Groups of one-day-old BALB/c suckling mice were inoculated intraperitoneally (i.p.) with the virus-sera and virus-PBS mixture. **(A)** Mice with different antiserum treatment at 11 days post-infection with EV71. The mouse on the left side received anti-chimeric VLPs sera and the one on the right side received anti-HBcAg VLPs sera. The appearance of limb paralysis in mouse is indicated by arrows. **(B)** Two representative mice in the PBS-treated group die at 7 days post-infection with EV71. **(C)** Survival rates were recorded daily after infection for 16 days. 10 mice were used for each group.

EV71 BrCr-TR strain was used for viral infection because of its high virulence in neonatal mice. Groups of one-day-old BALB/c suckling mice (*n* =10 per group) were inoculated intraperitoneally (i.p.) with the virus-sera mixtures that had been incubated overnight at 37°C. After 7 days, control mice receiving EV71 with either PBS or anti- HBcAg VLPs sera started to show symptoms, such as reduced mobility, limb weakness, limb paralysis, and death (Figure 7A and B). The survival rates were 20% and 40% for the PBS and anti-HBcAg VLPs sera recipient groups, respectively, at 16 day post-inoculation (Figure 7C). In contrast, 90% of mice treated with mixture of anti- chimeric VLPs sera remained healthy and survived throughout the course. These observations confirmed previous experiments using RD cells, that immune sera elicited by chimeric particles neutralized EV71 infection.

### Identification of “core sequence” by epitope mapping

**Figure 8:**
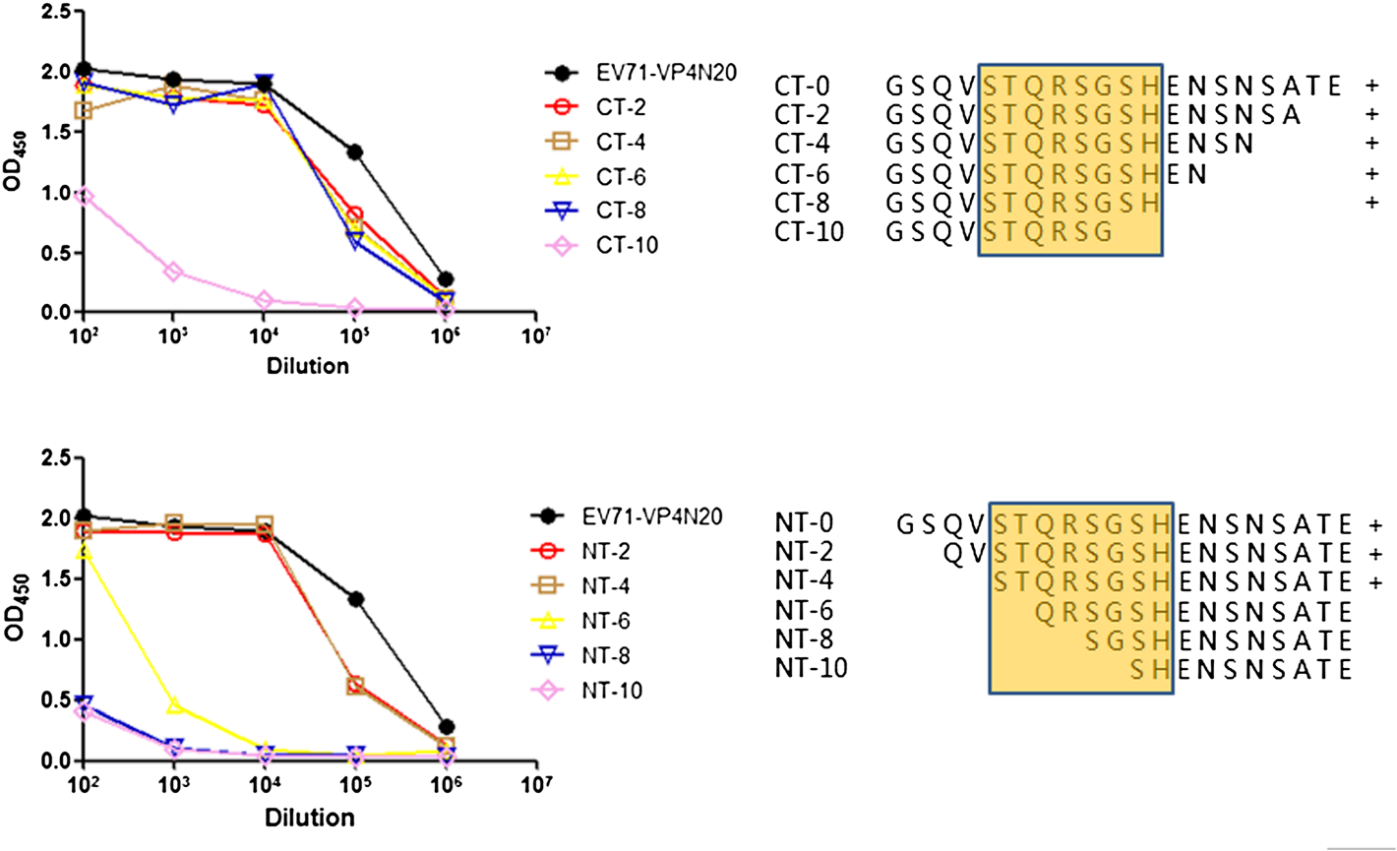
**Effects of peptide length on recognition of VP4 peptides by antibodies raised against the first N-terminal 20 residues of EV71 VP4.** The top panel shows the ELISA reaction of the polyclonal serum to peptides truncated at the carboxyl end of the 20-mer. The bottom shows the same with the truncations at the amino end, and the highlighted yellow region shows the minimal apparent “core” of the peptide for antibody recognition. The plus signs on the right of the diagram illustrate whether the polyclonal serum binds to the peptide fragment. OD450: optical density at 450 nm.

VP4N20 peptide can elicit neutralizing antibody and conferred cross-protection against EV71 strains belonging to different genotypes *in vitro*. We further investigated the most immunologically essential sequence of the peptide by epitope mapping experiments to find out the minimal peptide sequence showing the highest efficiency for inducing the production of neutralizing antibody. A panel of peptides corresponding to the N- and C-terminal truncations of VP4N20 peptide was used for epitope mapping. As shown in Figure 8, the polyclonal antibodies raised against the VP4N20 peptide were very sensitive to truncation of either end of the peptide. Once six (N-terminal) or ten (C-terminal) residues were clipped from either end of the inoculation peptide, the polyclonal antibodies were no longer able to bind. One interpretation of these results is that there is an essential “core” of the peptide that does not tolerate truncation. The “core sequence” is highly conserved amongst the VP4 sequences of EV71 strains from various genotypes based on the alignment data (Figure 1). Our results suggest that VP4N20 peptide may potentially elicit a pan-genotypic immune response once the right segment of VP4 is identified.

## Discussion

Gene mutation and genetic recombination were frequently observed during EV71 epidemics, resulting in substantial genetic variation of EV71 genome and the emergence of the various EV71 subgenotypes [21]. Virus variants which possess a selective advantage in terms of ability to evade host immune surveillance can spread and become established within human populations. EV71 is classified into 11 subgenogroups according to the genetic variation of VP1 gene [15]. EV71 genotype-related HFMD outbreaks were extensively reported previously. Genotype B1 was the major viral strain in circulation from 1970 to 1980 [22]. The co-circulation of four subgenotypes C1, C2, B3, and B4 were observed in Malaysia between 1997 and 2000 [22]. The genotypes B2, C4 and B5 were reported to be the circulating strains from 1998 to 2009 in Taiwan [22,23]. One exceptional case was observed in China, where genotype C4 was identified as the dominant viral strain responsible for the HFMD outbreak from 2007–2011 [24,25]. Thus, an ideal vaccine should elicit effective cross-neutralizing antibody responses against different genotypes of EV71.

Several different types of EV71 vaccine candidates have been investigated in animal model, including recombinant vaccines [3,26-28], peptide vaccines [19,20], live attenuated vaccines [29,30] and formalin-inactivated virion vaccines [31-34]. Only inactivated EV71 vaccines are being evaluated in human clinical trials due to its superior immunogenicity and more matured manufacturing technologies. Inactivated EV71 virion vaccines have been found to be able to elicit cross-neutralizing antibody responses against EV71 strains of different genotypes in mouse model [34]. However, constant genetic evolution has been observed in EV71 genome [35], the efficiency of protective immunity elicited by currently used inactivated EV71 virion vaccines against novel EV71 variants thus still remain to be evaluated. Several linear neutralizing epitopes have been identified in the VP1 and VP2 proteins of EV71, which are very helpful for detailed understanding of broad immunoprotection elicited by inactivated EV71 virion vaccines and peptide-based vaccine design [16,18-20].

Targeting conserved regions within the immunogens for vaccine development is an alternative approach to deal with high genetic diversity of pathogens. EV71 VP4 gene is more conserved than VP1, VP2 and VP3 genes. We therefore attempted to identify neutralization epitopes in VP4 gene. We found that the first 20 N-terminal amino acid residues are highly conserved amongst the VP4 sequences of EV71 strains from various genotypes. In the present study, the peptide consisting of first 20 a.a. at N-terminal of VP4 of EV71 genotype C4 (VP4N20) was fused to HBcAg protein. HBcAg particles have been extensively exploited as a carrier to improve the immunogenicity of foreign protein segments presented on their surface. As expected, the fusion proteins were able to assemble into chimeric VLPs in bacteria as efficient as unmodified HBcAg. Immunization of the chimeric VLPs was able to elicit VP4N20 specific antibody in mice. *In vitro* neutralization assay showed that antibodies raised against chimeric VLPs were able to not only neutralize EV71 of genotype C4 but also displayed a similar neutralizing activity against EV71 of genotype A, indicating that immunization of the first 20 N-terminal amino acids of VP4 of EV71 genotype C4 is able to elicit neutralizing antibody which exhibited a broad neutralizing activity against different genotypes of EV71 *in vitro*.

Neutralizing antibodies play an important role in the immune defense against picornavirus infection. In the case of poliovirus, antibodies raised against VP4 and the N termini of VP1 of poliovirus serotype I were capable of neutralizing the poliovirus virions [36,37]. Similar results were reported in the studies on rhinovirus, antibodies against the N-terminus of VP4 were found to successfully neutralize viral infectivity *in vitro*[38]. VP4 played a pivotal role during picornavirus cell entry despite the fact that VP4 is buried in the interior of the capsid at the capsid-RNA interface [39], indicating that the picornavirus capsid structure is more dynamic than the suggested crystal structure. It has been shown that the attachment of picornavirus on the receptor can trigger conformational alteration of virus and lead to the externalization of VP4 and the N-terminus of VP1 [40,41]. The egress of the myristylated VP4 is involved in the formation of channels responsible for the safe release of the picornavirus genome to the cell cytoplasm [42]. The exposure of VP4 to the outside of the capsid may potentially result in anti-VP4 antibody-mediated neutralization against picornavirus. However, our results on neutralizing responses elicited by N-terminus VP4 of EV71 are consistent with previous reports [38,42]. Furthermore, we identified the “core sequence” of N-terminus VP4 of EV71 responsible for antibody recognition. The “core sequence” is highly conserved amongst the VP4 sequences of EV71 strains from various genotypes and that of CA16. Whether antibody responses elicited by the N-terminus of EV71 VP4 are capable of neutralizing CA16 virions still remains to be investigated.

## Conclusions

In summary, this study identified an immunodominant epitope located at the N-terminal of EV71 VP4 protein. The fusion proteins of HBcAg and N-terminal of EV71 VP4-derived peptide were able to spontaneously assemble into chimeric VLPs. Mice immunization with these chimeric VLPs elicited neutralizing antibodies against EV71 of different genotypes. The “core sequence” responsible for immune stimulation was found to be highly conserved across different EV71 genotypes.

## Methods

### Plasmid constructions and bacterial strains

The peptide (VP4N20) that corresponds to first 20 residues at the N-terminal of VP4 of EV71 (Bj08) was inserted to HBcAg (HBc-N149) loop region between amino acids 78 and 79. The fusion protein was named as HBc-N149-VP4N20. To construct the plasmid expressing the fusion protein, DNA fragment encoding HBc-N149-VP4N20 was synthesized and amplified using primers P1u (5'- CCGCTCGAGCACCACGGTGGTT-3') and P1d (5'- GGAATTCCATATGGATATTGATCCGTATAAAG-3'). The PCR products were double-digested by *XhoI* and *NdeI* and subsequently inserted into the vector pET22b(+) (Novagen, USA). DNA fragment encoding HBc-N149 was amplified by using the primers P1u, P2d (5′-TGGGCAGCAATCTGGAAGATCCGGCGAGCCGCGAACTG-3′), P2u (5′- ACCAGTTCGCGGCTCGCCGGATCTTCCAGATTGCTGCCCA-3′) and P1d by using HBc-N149-VP4N20-encoding gene as a template and further inserted into the vector pET22b (+). The accuracy of the constructs was confirmed by sequencing. *E. coli* strain BL21 (DE3) (BeiJing TIANGEN BIOTECH, China) were used for protein expression.

### Expression and purification of recombinant proteins

Overnight cultures of BL21 (DE3) cells harboring the recombinant plasmids were diluted 1:400 in 1 L of LB broth containing 100 μg/ml ampicillin, and grown until reaching an OD_600_ of 0.4-0.6. Protein expression was then induced by 0.1 mM of isopropyl-β-d-thiogalactopyranoside (IPTG). After shaking at 37°C for 5 h, the bacteria were collected by centrifugation at 12,000 rpm for 10 min at 4°C, and the pellets were resuspended in 100 ml of balance buffer (pH 8.0, 50 mM Tris, 100 mM NaCl, 10 mM imidazole). For protein purification, the bacterial cells were lysed by ultrasonication, followed by centrifugation at 13,000 rpm for 15 min at 4°C to remove bacterial debris. The clear supernatant was applied to a Ni Sepharose column (GE Healthcare Life Sciences, USA) according to the manufacturer’s recommendations. The columns were washed with washing buffer (pH 8.0, 50 mM Tris–HCl, 100 mM NaCl, 50 mM imidazole,) and bound proteins were eluted with elution buffer (pH 8.0, 50 mM Tris–HCl, 100 mM NaCl, 200 mM imidazole). The peak fractions were collected and analyzed by SDS-PAGE. The purity of the samples was determined by densitometric scanning. The proteins were dialyzed to PBS buffer (pH7.4) for 8 hours at RT and examined by electron microscopy and used for immunology studies.

### SDS-PAGE and Western blotting

Electrophoresis was performed in 12% SDS polyacrylamide gels and the recombinant proteins were detected by Western blotting using a monoclonal antibody (mAb) against the polyhistidine (His) tag in the C-terminal region of the fusion protein. Briefly, the transferred PVDF membrane was blocked with 2% (w/v) BSA in TBS for 1 h at 37°C, and washed thrice with TBS – 0.05% (v/v) Tween 20, then the membrane was incubated with a 1:5,000 dilution of anti-His tag (mouse mAb, CWBIO, Beijing, China) in a 0.2% BSA-TBS – 0.05% Tween 20 solution for 1 h at 37°C, and washed thrice with TBS – 0.05% Tween 20. Protein bands were probed with 1:2,000 dilution of HRP-conjugated goat anti-mouse IgG (CWBIO, Beijing, China) and washed thrice as described above. Chemiluminescence was applied as instructed by the manufacturer (Li-COR Odyssey, USA).

### Electron microscopy

The formation of HBcAg VLPs and chimeric VLPs (HBc-N149-VP4N20) was analyzed by negative staining electron microscopy according a previously described method [3]. Briefly, proteins were adsorbed to 230 mesh carbon-coated copper grids and incubated for 1 min. The grids were then washed once with PBS and stained for 45 s with 2% phosphotungstic acid. Specimens were evaluated using an electron microscope (H-7650, HITACHI, Japan).

### Immunization of animals

Pathogen-free female BALB/c mice were purchased from Beijing HFK Bioscience Co. (Beijing, China). All animals were housed at pathogen-free conditions. Animal experiments were performed in accordance with current guidelines for the Care and Use of Laboratory Animals of Experimental Animal Center of Military Medical Sciences and approved by the center. For mice experiments, five female BALB/c mice (6–8 weeks) per group were vaccinated intramuscularly (i.m.) with recombinant proteins HBc-N149 (5 μg/mouse) or HBc-N149-VP4N20 (5 μg/mouse) at week 0. The second injection was performed at week 3. QuickAntibody™ from KBQ Biotechnology Co. (Beijing, China) was used as an adjuvant. Control group was immunized with PBS plus adjuvant. The immunized animals were bled at week 0, 2, 5, 8 for antibody detection.

### ELISA

Direct ELISA was used for detection of antibodies in the sera of immunized animals. The peptide VP4N20 was synthesized by Scilight-Peptide (Beijing, China) and conjugated with Bull Serum Albumin (BSA-VP4N20). The peptides were purified using high-pressure liquid chromatography. ELISA plates (96-well) were coated with 250 ng/well of BSA-VP4N20 in coating buffer (50 mM Na_2_CO_3_–NaHCO_3_, pH 9.6) overnight at 4°C. After washing with PBS-0.05% (v/v) Tween 20 thrice, the plates were blocked with 2% (w/v) BSA in PBS for 2 h at 37°C. Sera were tested at 2-fold serial dilutions starting at 1:100. The plates were incubated at 37°C for 1 h and washed thrice with PBS-0.05% Tween 20. HRP-conjugated goat anti-mouse IgG (CWBIO, Beijing, China) was then added into each well in a 1:2000 dilution, and incubated at 37°C for 1 h. The plates were washed thrice and developed with TMB solution (Tiangen Biotech, Beijing, China) in a dark room for 15 min, and the enzyme reaction was stopped by adding 2 M H_2_SO_4_. The absorbance at 450 nm was measured using a microplate reader (Bio-Rad, USA).

### Epitope mapping

Enzyme-linked immunosorbent assay (ELISA) protocols were used for all the epitope mapping experiments. Peptides truncated at the carboxyl end or the amino terminus were purchased from Scilight-Peptide (Beijing, China). The peptides were conjugated with Bull Serum Albumin (BSA). The purity was higher than 90%.

### In vitro neutralization assay

EV71 BJ08 (genogroup C4) and BrCr-TR (genogroup A), were propagated in RD cells. Virus titers were determined using RD cells by the microtitration method and expressed as the 50% tissue culture infective dose (TCID50) according to the Reed–Muench method. Two-fold serial dilutions of sera were prepared using Minimum Essential Medium (MEM,Gibco®) containing 2% FBS. The EV71 stock was diluted to a working concentration of 100 TCID50/50 μl. The neutralization assay was conducted using 96-well plates. In each well, 50 μl of diluted serum sample was mixed with 50 μl of EV71 at 100 TCID_50_, and incubated overnight at 37°C. Next, 100 μl of cell suspension containing 10,000 RD cells was added to wells containing the virus/antiserum mixtures and incubated at 37°C. After 7 days, the cells were observed to evaluate the appearance of cytopathic effects. Neutralization titer was defined as the highest serum dilution that could completely protect cells from developing cytopathic effects.

### Mouse protection assay

To evaluate protective efficacy of the immunized sera against EV71 infection, *in vivo* infection experiments were performed. Briefly, 50 μl of sera or PBS were incubated with 10 LD_50_ of EV71 BrCr-TR (50 μl in sterile RD cell supernatant) at 37°C for 2 hour. Groups of one-day-old BALB/c suckling mice (n = 10 per group) were inoculated intraperitoneally (i.p.) with the virus-sera mixture or virus-PBS mixture. All mice were monitored daily for clinical symptoms and death for up to 16 days after inoculation.

## Competing interests

The authors declare that they have no competing interests.

## Authors’ contributions

The authors’ contributions to this research work are reflected in the order shown. MZ contributed to the majority of experimental works and the writing of the manuscript. YB and WL carried out protein expression and purification. YH and XX participated in virus preparation and their characterization. SC participated in in vivo neutralization assay. WS and XS directed the research, designed and coordinated the project, analyzed the data, and wrote the manuscript. PC and YZ conceived the study and participated in its design. All authors read and approved the final manuscript.
